# Mass media exposure and use of reversible modern contraceptives among married women in India: An analysis of the NFHS 2015–16 data

**DOI:** 10.1371/journal.pone.0254400

**Published:** 2021-07-13

**Authors:** Ranjita Ghosh, Arupendra Mozumdar, Aparajita Chattopadhyay, Rajib Acharya

**Affiliations:** 1 Department of Development Studies, International Institute for Population Sciences, Mumbai, India; 2 Reproductive Health Division, Population Council, New Delhi, India; University of Botswana, BOTSWANA

## Abstract

Since the inception of the National Programme for Family Planning, messages on family planning (FP) have been promoted across India using different mass media platforms. Mass media plays an important role in disseminating important information among the masses, such as how reversible modern methods give women more reproductive choices than opting for permanent methods that limit their child-bearing capacity. Mass media can provide a continuous flow of information and motivation to deter women from discontinuing the methods they have opted for. However, very few studies have been conducted on this issue, especially using recently available data. This study particularly focuses on exposure to mass media and the use of reversible modern methods of family planning among married women in India. The data for this study was obtained from the National Family Health Survey (2015–16) on currently married women aged 15–49 years. The association of reversible modern method use with media exposure variables was examined, controlling for a set of independent variables from multiple levels—individual, district, state, and region. The findings from this study showed that television was the most important medium for disseminating information on FP among married women in India. Spatial analysis revealed that some districts in the north, parts of the northeast, and Kerala in South India lacked any television exposure. The results from the decomposition analysis showed that mass media exposure was associated with a 14% increase in the use of reversible modern methods. Results from the multilevel analyses showed that exposure to TV along with other media (AOR 1.57 95% CI 1.49–1.65) and exposure to FP messages through different media (AOR 1.22 95% CI 1.12–1.32) had a significant positive effect on the use of reversible modern methods even when various individual, district, state, and regional-level factors were controlled. The findings of this paper provide evidence supporting the use of mass media to promote and increase awareness of voluntary contraceptive use in India. An increase in mass media exposure coupled with improvement in coverage and services of the FP program can significantly increase the use of reversible modern methods in a cost-effective yet efficient manner among women in need of FP services.

## Introduction

After launching the National Programme for Family Planning in 1952, India became the first country in the world to implement the largest national-level government-sponsored FP program [1]. India’s National Family Welfare Programme has been raising awareness on FP and its benefits using multiple media channels [2]. These awareness campaigns have positively impacted people’s attitudes and beliefs towards FP and its acceptance [2].

The primary objective of India’s FP program was to stabilize the country’s population, which was rapidly increasing in the early 1950s. With time, the program has undergone many changes, and now, along with the goal to stabilize the population, it also promotes reproductive health to reduce maternal, infant, and child mortality and morbidity. The inclusion of reproductive health by promoting different choices of contraceptive methods was an important step taken by the government. It not only focused on controlling the increasing population but also on improving the health of women, thereby empowering women in India. Mission Parivar Vikas, launched in 2016, aimed for improved access to contraceptives and FP services in high fertility districts spreading over seven high focus states of Assam, Bihar, Chhattisgarh, Madhya Pradesh, Rajasthan, Jharkhand, and Uttar Pradesh. It focused on using spacing methods for FP by introducing two new reversible contraceptive methods. The main goal was to reduce India’s overall fertility rate to 2.1 by 2025.

Within the FP program, mass media campaigns and FP messaging focused on providing information about the program as well as educating women on the advantages of small family norms and the use of contraceptives. Messages through mass media help disseminate information on various issues to different sections of society in a much simpler manner. This would not have been possible otherwise, especially in the context of uneducated women, who currently comprise 33% of married Indian women [3]. Exposure to mass media also helps women make their own decision regarding FP use [2].

Earlier research studies have demonstrated that mass media has the potential to influence the behavior of an individual. FP messages that are being promoted by mass media influence complex issues like contraceptive use among women. Westoff and Rodriguez conducted a study on the role of mass media and FP in Kenya [4]. Their study found a strong association between women’s reporting of hearing or seeing FP messages with contraceptive and reproductive preferences—even after controlling for a variety of lifestyles, urban and rural residential dwelling, and socioeconomic variables. Women who were not exposed to any messages reported an average of 5.5 children as their ideal family size, while those who were exposed to three types of messages reported 4.7 children as ideal family size—suggesting mass media could have an important effect on reproductive behavior.

Retherford & Mishra worked on mass media exposure and contraceptive use based on data from the National Family Health Survey (NFHS) 1992–1993 [2]. The results of their study suggested that general exposure to electronic mass media had a positive impact on the use of contraceptives among women. A study conducted in the Union Territory of Goa on the exposure to mass media and its effect on FP methods used by women concluded a positive association between mass media exposure and the FP methods are chosen [5]. Further, the association between multimedia behavior change communication (BCC) campaigns and women’s and men’s use of and intention to use a modern contraceptive method were studied in target areas of Uganda [6]. The results of the study indicated that exposure to BCC messages was associated with increased contraceptive use and intention to use. A similar study from urban areas of Kenya, Nigeria, and Senegal reported an association between men’s exposure to FP demand-generation and the use of modern contraceptive methods [7]. The study also highlighted that in Kenya, those who participated in Urban Reproductive Health Initiative (URHI)-led community events had nearly four times higher odds of reporting the use of modern methods, while in Senegal, being exposed to URHI television programs and listening to a religious leader’s speech favoring FP were associated with the use of modern contraceptive methods.

Data from the 2005 Indian Human Development Survey reported that mass media exposure affected the adoption of contraceptives across the poverty line [8]. The study found that watching television had a strong effect on contraceptive use. Using DHS data, a study from Nigeria found that newspaper readership was significantly associated with contraceptive use among families living above the poverty line but not significantly associated among families living below the poverty line [9]. The study also examined the effect of spatio-demographic variables on the relationship between mass media messages and the use of FP methods. The study was conducted in all 36 states of Nigeria and Abuja, and reported significant variations within spatio-demographic groups in access to mass media messages and the use of FP. The study also showed that access to mass media messages increased the likelihood of using FP.

A study on determinants of contraceptive use in Orissa revealed that the probability of using contraception among women who had been exposed to any mass media was significantly higher among women with two, three, and more than three living children [10]. Meekers et al. found that radio communication campaigns had a significant effect on the ever use of condoms in Malawi [11]. A study by Modugu et al. showed that education focused entertainment shows led to a significant increase in the uptake of FP methods in rural Bihar and Orissa [12].

One study demonstrated that mass media was men’s primary source of reproductive health information, although they expressed interest in getting information through discussions with knowledgeable sources [12]. Exposure to mass media is related to the use of prenatal care services even when other likely causes of the relationships are statistically controlled [13]. A study on trends, patterns, and determinants of long-acting reversible contraceptive (LARC) methods among women in sub-Saharan Africa showed significant predictors for the uptake of LARC methods, including fertility-related characteristics, age, level of education, work status, wealth index, and exposure to mass media [14]. Another study on LARC conducted in Nepal found younger women’s age, low or no husband’s education, from an indigenous community, and being in the lowest wealth quintile negatively influenced the use of LARC; while women with husbands as skilled workers, parity less than two, and desire of having future children positively influenced the use of LARC [15]. Using multilevel analyses, many studies examined the association of contextual variables on reproductive, maternal, and child health including FP indicators [16, 17].

The literature demonstrated that mass media is important for the promotion as well as the dissemination of information on different FP methods among the masses which results in higher contraceptive use. Reversible methods give women more reproductive choices than opting for permanent methods which limit their child-bearing capacity. Information on reversible contraceptive use needs to be regularly reinforced to motivate use and decrease discontinuation. Mass media can play an important role in increasing awareness, intention, and use of reversible modern methods by providing a continuous flow of information and motivation to retain women as contraceptive users.

To fill the research gap highlighting the role of media in FP programming, especially to promote the use of reversible modern methods, this research aimed to study the level of mass media exposure and use of reversible modern contraceptives, the spatial variation of mass media exposure, use of reversible modern contraceptives, and the association of mass media exposure and use of reversible modern contraceptives by controlling programmatic, demographic, socio-economic, and other contextual factors among married women in India. The findings may be useful in strengthening ways of improving exposure to different mass media channels in order to increase the use of FP in India.

## Materials and methods

### Ethics statement

The NFHS 2015–16, the survey data we have used in this paper, received ethical clearance from the Ethical Review Board of the International Institute for Population Sciences, Mumbai, India. The interviewers obtained informed consent from each respondent before the interview and made their best effort to ensure privacy. The data are available in the public domain without any personal identifier.

### Contraceptive usage in India

Around 54% of married Indian women were currently using any contraceptive methods in 2015–16, and about 48% were using any modern contraceptives [3]. About 75% of those who were currently using any modern contraceptive were using sterilization. Among the reversible modern contraceptives, current usage was highest for condoms (6%), followed by pills (4%) and intrauterine contraceptive devices (2%). The current use of modern contraceptives was high in southern states of the country, while the usage is much lower in northern and northeastern states.

### Conceptual framework

**Fig 1:**
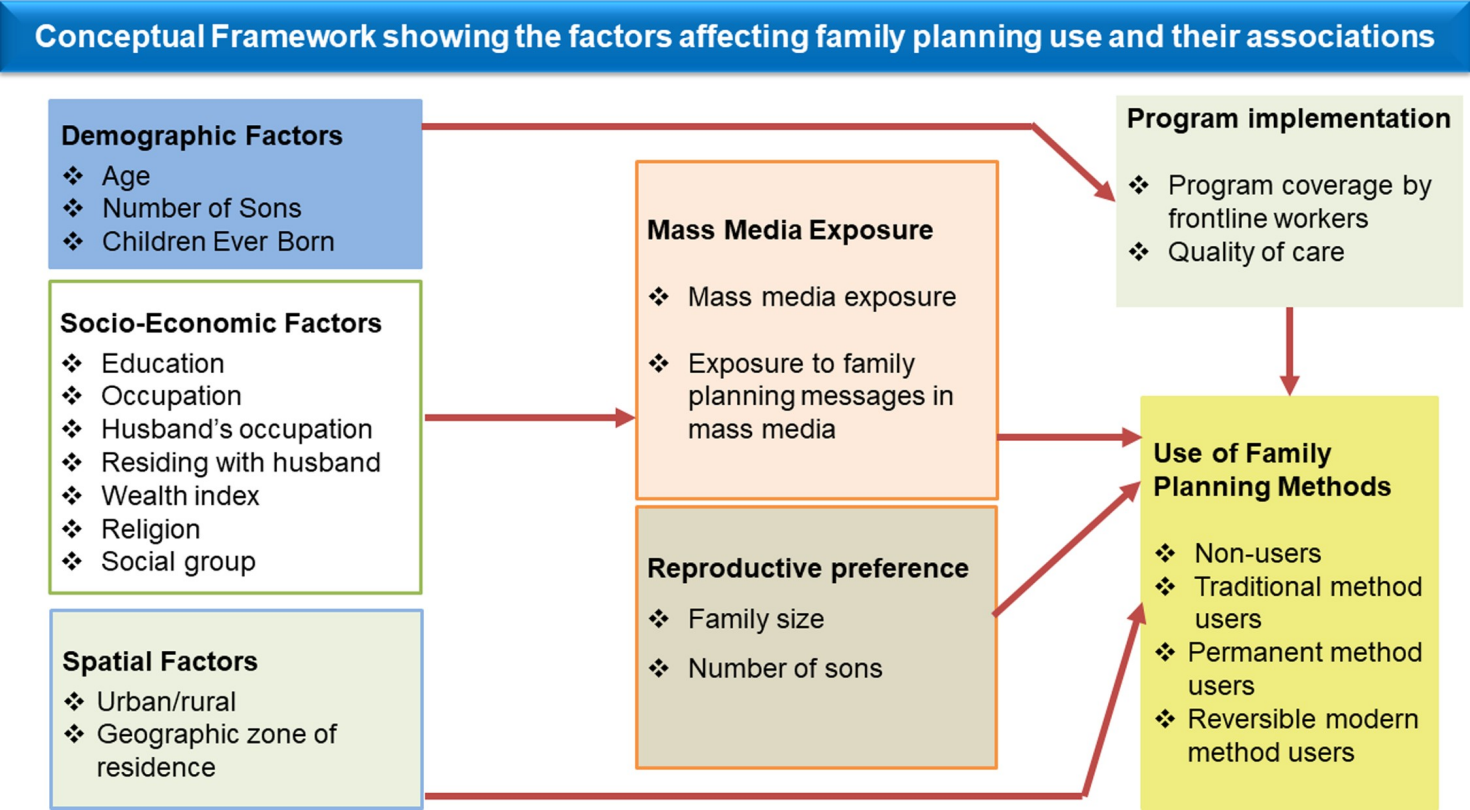
Conceptual framework showing the associations of mass media exposure, background characteristics of women, and other determining factors.

The analyses for this study were conducted based on a conceptual framework given in Fig 1. The framework was developed using the evidence generated based on existing literature on mass media and the use of FP methods [5–7, 14–19]. The framework showed pathways through which different factors, including exposure to mass media, could affect the use of FP methods. Through the framework, the analyses also illustrated the inter-relationship between mass media exposure and other individual and contextual variables. It was hypothesized that the use of FP was affected by various factors at four different levels: individual, district, state, and region. The list of variables considered for each level is provided later in this section. It was assumed that the individual factors could either directly affect the use of FP or through mass media exposure and other contextual variables.

### Data source and extracted samples

The data for this study were obtained from NFHS 2015–16 [3]. The survey was carried out by the International Institute for Population Sciences, Mumbai under the financial support of the Ministry of Health and Family Welfare, Government of India. For the first time, the NFHS 2015–16 was designed to provide the estimates of all the key indicators at the national, state (29 states and 6 union territories), and district levels (for 640 districts).

The NFHS 2015–16 sample is a stratified two-stage sample. The 2011 census served as the sampling frame for the selection of PSUs. PSUs were villages in rural areas and Census Enumeration Blocks (CEBs) in urban areas. In every selected rural and urban PSU, a complete household mapping-and-listing operation was conducted prior to the main survey. Selected PSUs with an estimated number of at least 300 households were segmented into segments of approximately 100–150 households. Two of the segments were randomly selected for the survey using systematic sampling with probability proportional to segment size. Therefore, an NFHS 2015–16 cluster is either a PSU or a segment of a PSU. In the second stage, in every selected rural and urban cluster, 22 households were randomly selected with systematic sampling. The details of the sampling design and survey procedure of the NFHS 2015–16 are available in the survey report [3].

For this study, data on married women aged 15–49 years were extracted from the women’s file of NFHS 2015–16. The total number of married women considered for this study was 499,627 and we have used this sample to examine the association between exposure to mass media and contraceptive use in general. For the final analysis—examining the relationship of exposure to mass media and the current use of reversible modern contraceptive—women or their husbands who had undergone sterilization, hysterectomy, were in menopause, or infecund were dropped. After these exclusions, the final sample for this study was 249,635. The step-by-step exclusion of the sample is presented in S1 Fig.

### Dependent variables

Current usage of contraceptive methods by women was coded into four categories: not using any method, using traditional methods, using permanent methods, and using reversible modern methods. The dependent variable for this study was the current usage of reversible modern contraceptive methods by women.

### Independent variables

The independent variables for this study were taken from data at different levels: individual, district, state, and region. The individual-level variables consisted of exposure to mass media, exposure to messages on FP in media, and other background characteristics of the women.

Exposure to mass media is a composite variable that included different combinations of the frequency of listening to the radio, watching television, and reading newspapers/magazines. In NFHS 2015–16 data, the frequencies of exposure for each type of mass media were recorded as not at all, sometimes, or daily. After examining the frequency distribution of the responses from the respondent, a composite variable was created with four categories: not exposed, only exposed to television viewing (TV), exposed to TV and other mass media, and exposed to other mass media excluding TV.

The second individual-level variable—exposure to messages on FP in media—was also a composite variable of whether the respondent heard FP messages through radio, television, newspaper, or poster/hoarding. Other individual-level independent variables included women’s age, age at marriage, place of residence, education level, husband’s education level, occupation, husband’s occupation, husband’s residential status, social group, religion, wealth index, children ever born, and the number of living sons. The categorization of all independent variables has been presented in S1 Table. The missing values were dropped from the analysis. The missing values related to the variable on caste/tribe were recoded as ‘others’.

All variables might not have the same effect at every level. It was assumed that the contextual variables could have also influenced the outcome. The contribution of mass media was unclear based on the individual-level independent variables, due to which multilevel analyses were conducted at the district, state, and regional levels. The district-level variable was the outreach coverage of the FP program. This indicator was measured by the percentage of never users of contraceptives who discussed FP with health workers. Non-users were taken into consideration as it would help in determining whether they were informed on FP or not. Moreover, informing them about FP use would increase their knowledge about different methods, leading to potential use in the future. The percentage of women who received complete information to make an informed choice—measured in terms of method information index (MII)—was considered as a state-level variable. MII represented the percentage of current modern contraceptive users who reported whether the provider informed them about other methods, side effects; and if informed of side effects, what they needed to do [20]. MII was used as one of the core indicators of informed choice in many global projects monitoring the progress of FP programs, such as FP 2020, Track 20, Performance Monitoring for Action, etc. For this analysis, MII was calculated among reversible modern method users for each Indian state and used as a state-level variable. The geographic region of the respondent’s household was considered a variable for the regional level. No fixed-effect variable was used for controlling the effect at the regional level.

### Statistical analysis

Univariate analysis was conducted for currently married women to show the percentage of exposure to mass media and the exposure to FP messages. Bivariate analysis was conducted to examine the association of exposure to mass media and FP messages with the use of FP methods among currently married women.

Choropleth maps were created to show spatial variation. District-wise maps were created to show the spatial variation for the percentage of exposure to television, usage of reversible methods, and health workers who shared information on FP use with never-users. The state-wise distribution of MII was also shown separately. The lighter shades in the map show lower values while the darker shades indicate higher values in percentage.

#### Decomposition analysis

Fairlie decomposition was used to study the contribution of mass media exposure to the use of reversible modern methods. Reversible modern method use was considered as the dichotomous dependent variable (coded as 0 = non-users or traditional method users, 1 = users of reversible modern methods), and mass media exposure (coded as 0 = non-exposed, 1 = exposed) was considered as the group variable for the analysis. Women who were infecund, in menopause, had a hysterectomy, postpartum amenorrhea, or had undergone sterilization were excluded from the analysis. For this analysis, the independent variables were converted into dichotomous variables, which included age (0 = 15–24, 1 = >24), place of residence (0 = rural, 1 = urban), education qualification (0 = not educated 1 = literate), caste (0 = scheduled caste, scheduled tribe, or other backward classes; 1 = others), religion (0 = Hindus, 1 = non-Hindus), wealth index (0 = poor, poorer, and middle; 1 = rich, and richer), number of sons (0 = no son, 1 = 1+son), and children ever born (0 = no child, 1 = one or more children). Percentage of each independent variable and total contribution of mass media in contraceptive use was calculated using the following formula:

Percentagecontribution=(Coefficientoftheindependentvariable/Totalexplained)*100

Thetotalcontributionofindependentvariablesonreversiblecontraceptiveuse=(Totalexplained/Difference)*100

#### Multilevel analyses

Since independent variables for these analyses came from more than one level, multilevel analyses were used to identify the predictors of FP use. The levels used in the model included individual, district, state, and regional levels. The large sample size of the NFHS 2015–16 allows us to get the four-level data structure i.e. individuals are nested within a district, districts are nested within a state, and states are nested within a region.

A logistic regression model was designed involving four levels (individual, district, state, and region), which can be written as follows:

$$Log\left(\frac{\pi_{ijkl}}{1-\pi_{ijkl}}\right)=Y_{ijkl}=\alpha +\beta X_{ijkl}+\gamma Z_{jkl}+\delta W_{kl}+\phi U_{l}+r_{0l}+s_{0kl}+d_{0jkl}+e_{0ijkl}$$ (1)

where *Y*_1_*ijkl* = the current use of modern reversible methods for individual *i* in *j* district of state *k* in *l* region; α = constant; *X*_*ijkl*_, *Z*_*jkl*_, *W*_*kl*_, and *U*_*l*_ are vectors of variables; *β*, *γ*, *δ*, and *ϕ* are regression coefficients; and *e*_*0ijkl*_, *d*_*0jkl*_, *s*_*0kl*_, and *r*_*0l*_ are residuals at the individual level, district level, state level, and regional level, respectively.

Three different models were used for these analyses, with the dependent variable being women using any reversible modern method (coded as 0 = non-users and traditional methods, 1 = reversible modern methods). The women who were infecund, in menopause, had a hysterectomy, had post-partum amenorrhea, and had undergone sterilization were excluded from the analyses. The analyses excluded permanent method users as exposure to mass media FP messaging would not affect their status of being a user. The analyses started with a null model i.e., Model-0, followed by three subsequent models using cumulative sets of independent variables. For Model-1, media-related variables were considered as independent variables. In Model-2, variables related to background characteristics and reproductive history were added along with the variables from Model-1. In Model-3, variables at the district and state levels—FP program coverage and the percentage value of MII—were added as independent variables along with the variables in the previous models. The highest level of the model was the geographic regions comprising of 6 geographic groups of states, as it was expected that the distribution of both dependent and independent variables had some spatial patterns across different geographies.

Adjusted odds ratios (AOR) with 95% confidence interval (CI) were reported to show the fixed effects of the explanatory variables. To show the random effects, variance partition coefficients (VPC) and proportional change in variance were reported for the individual, district, state, and regional levels. The formulae are shown below:

$$VPC_{n}=\frac{VAR_{n}}{\left\{\sum_{n=2}^{N}VAR_{n}+\frac{\pi^{2}}{3}\right\}}$$ (2)

Where, *VPC*_*n*_ is the VPC at the *n*th level while *VAR*_*n*_ is the variance at the *n*th level of regression. Here, the highest level is represented by *n*.

$$PCV_{l}=\frac{\left(VAR_{ln-}VAR_{li}\right)}{VAR_{ln}}$$ (3)

Where, *PCV*_*l*_ is the PCV of a level, *VAR*_*ln*_ is the variance of the null model, and *VAR*_*li*_ is the variance of the model at a specific level.

All the statistical analyses were conducted using STATA 15 software. Choropleth maps were prepared using Arc Map 10.3 software and multilevel analyses were conducted using the “runmlwin” program to run the MLWin software within STATA.

## Results

### Exposure to mass media, messages on FP

The univariate analysis revealed that among married women of reproductive age (15–49 years), 21% were not exposed to any mass media; 38% were exposed to television viewing, followed by television in combination with ‘other’ types of mass media (38%) such as radio and newspaper/magazine, and 3% of women used only ‘other’ mass media and were not exposed to television viewing. About 8% of married women were exposed to all types of mass media.

Bivariate analyses showed that the majority of respondents were not (about 86%) listening to the radio, about 60% was watching television daily, about one-fifth of them were reading newspapers/magazines ‘sometimes’ in a week. Only about 12% of the respondents were reading newspapers/magazines daily (Table 1). There are significant variations in exposure to different mass media by background characteristics or women. An 11%-point difference was found in daily television viewing between the age group of 15–19 years and 25–29 years and a 9%-point difference in daily newspaper/magazine reading between 15–19 years and 40–44 years. The exposure to radio was significantly high among women with higher education (about 25%), and among those who were living in urban areas (18%). Television viewing and education level had a strong association—87% of women with higher education watched television daily, while only 36% of the respondents with “no education” did so. Similarly, more than half of the respondents with higher education read newspapers/magazines daily, while only 2% of the respondents with primary education read newspapers/magazines daily. Women living in urban areas had higher exposure in all forms of mass media—a 5%-point difference in radio listening between urban and rural, a 16%-point difference in television viewing, and a 28%-point difference in newspaper/magazine reading. Exposure to different forms of mass media was significantly higher among women from ‘rich’ families—women from ‘rich’ families had a 7%-point higher radio listenership, a 47%-point higher television viewership, and a 48%-point higher newspaper/magazine readership than women from ‘poor’ families. Overall, exposure to different forms of media was significantly higher among women living in urban areas, and those from ‘rich’ families, ‘other’ social groups, ‘Christian’ or ‘other’ religion, and living in southern states of the country. The results of chi-square tests showed highly significant associations (p<0.001) of exposure to all types of mass media with background characteristics of Indian women.

**Table 1:** Exposure to mass media by background characteristics for married women aged 15–49 years in India, NFHS 2015–16.

	Radio			Television			Newspaper/magazine			
Background characteristics	No	Sometimes	Daily	No	Sometimes	Daily	No	Sometimes	Daily	Total (N)
	%	%	%	%	%	%	%	%	%	
**All India**	85.7	10.5	3.9	24.3	15.9	59.9	65.1	22.7	12.2	4,99,627
**Age groups, in years**^1^^,^^2^^,^^3^										
15–19	86.8	10.3	3.0	30.8	18.5	50.8	67.0	28.2	4.8	18,067
20–24	85.9	10.9	3.2	24.5	16.5	59.0	63.0	28.6	8.4	78,431
25–29	85.5	10.7	3.8	23.1	15.0	62.0	60.5	26.9	12.6	1,00,355
30–34	85.4	10.6	4.0	23.4	15.4	61.3	62.3	24.1	13.7	88,810
35–39	85.5	10.4	4.2	24.2	15.6	60.2	66.1	20.2	13.7	82,250
40–44	85.4	10.2	4.4	24.6	16.2	59.2	68.7	17.4	13.9	68,580
45–49	86.2	9.7	4.1	24.9	16.3	58.8	73.1	14.2	12.7	63,135
**Education level**^1^^,^^2^^,^^3^										
No Education	90.4	7.7	1.9	45.3	18.9	35.8	99.2	0.7	0.1	1,64,986
Primary	87.9	9.4	2.8	24.8	19.5	55.8	83.9	14.2	2.0	71,376
Secondary	83.9	11.6	4.6	12.5	14.0	73.5	44.7	39.9	15.5	2,12,569
Higher	74.6	16.2	9.2	4.3	8.7	87.0	13.3	34.2	52.5	50,695
**Place of residence** ^1^^,^^2^^,^^3^										
Urban	82.0	12.2	5.8	8.0	10.6	81.3	46.5	28.9	24.6	1,66,944
Rural	87.5	9.6	2.9	32.4	18.5	49.1	74.4	19.6	6.0	3,32,683
**Wealth index**^1^^,^^2^^,^^3^										
Poor	89.1	8.7	2.2	51.2	21.8	26.9	88.5	10.4	1.1	1,89,316
Middle	86.7	9.9	3.34	14.6	16.8	68.5	71.3	23.9	4.8	1,02,278
Rich	82.0	12.3	5.7	4.4	9.9	85.7	40.8	33.2	26.0	2,08,033
**Caste**^1^^,^^2^^,^^3^										
SC	87.2	9.5	3.3	26.0	16.0	58.0	73.6	19.0	7.4	1,01,028
ST	87.8	9.7	2.5	38.4	21.6	40.0	80.8	14.5	4.7	45,537
OBC	85.8	10.3	3.9	24.9	15.3	59.8	65.0	23.1	11.9	2,17,677
Others	83.6	11.7	4.7	17.1	14.7	68.2	53.5	27.6	18.9	1,34,685
**Religion**^1^^,^^2^^,^^3^										
Hindu	85.5	10.5	4.0	23.6	15.6	60.8	65.0	22.7	12.3	4,06,897
Muslim	86.5	10.2	3.3	33.0	17.6	49.5	71.0	20.4	8.6	65,761
Christian	83.3	11.4	5.2	14.6	15.1	70.3	48.8	26.7	24.6	11,110
Others	88.4	8.8	2.8	12.7	15.0	72.2	54.0	30.2	15.9	15,859
**Region**^1^^,^^2^^,^^3^										
North	86.7	10.3	3.0	16.4	16.7	66.9	61.4	25.4	13.3	67,049
South	83.1	10.4	6.5	6.6	7.3	86.0	53.0	26.1	20.9	1,15,463
East	87.2	9.9	2.9	39.9	17.8	42.3	76.0	18.0	5.9	1,15,438
West	85.0	11.2	3.8	15.2	16.5	68.3	55.6	29.1	15.3	72,022
Central	86.3	10.6	3.1	35.7	20.4	43.9	73.3	18.5	8.1	1,12,719
Northeast	86.5	10.8	2.7	30.8	24.8	44.4	73.2	21.0	5.8	16,936

^1^ Significant association between independent variables and radio listening (Chi-square test, p<0.001)

^2^ Significant association between independent variables and television viewing (Chi-square test, p<0.001)

^3^ Significant association between independent variables and newspaper reading (Chi-square test, p<0.001)

Among married women aged 15–49 years who belonged to households that did not have a radio or TV, only 21% were not exposed to FP messages. Only 2% of women were exposed to FP messages among those households that had a radio. The percentage of exposure to FP messages increased to 57% for households with a TV. Among households that had both radio and TV, only 19% of women were exposed to FP messages (S2 Table).

#### Association of FP use with background characteristics

About 55% of currently married Indian women of 15–49 years were currently using any contraceptive methods, about 36% were using permanent methods, while only about 12% were currently using reversible modern methods (Table 2). Bivariate analyses showed that the current use of reversible modern methods was higher among women of 20–34 years of age. The current use of a reversible modern method among women with no education was only 6%; however, 21% of women with higher education were currently using reversible modern methods. Women living in urban areas, from ‘rich’ families, belonging to ‘other’ social groups, and ‘other’ religious groups were currently using reversible modern methods in higher proportions than their counterparts. In Northeastern and Northern India, the current use of reversible modern methods was high (25% and 19%, respectively) and television viewing was 69% and 83%, respectively. Although the current use of any modern contraceptive was highest among women of South India, the current use of reversible modern methods was just 2%. The current use of reversible modern methods was highest among women in Northeast India (25%), followed by North India (19%) and East India (14%).

**Table 2:** Use of family planning methods by background characteristics for married women aged 15–49 years in India, NFHS 2015–16.

	Family planning use				
Background characteristics	Non-users	Traditional methods	Permanent methods	Reversible modern methods	Total (N)
	%	%	%	%	
**All India**	**46.5**	**5.8**	**36.3**	**11.5**	**499,627**
Age groups in years*					
15–19	85.2	4.9	0.9	9.1	18,067
20–24	71.1	5.4	9.12	14.4	78,431
25–29	52.0	6.1	25. 9	16.0	100,355
30–34	37.8	6.5	40.9	14.8	88,810
35–39	32.8	6.8	49.6	10.8	82,250
40–44	34.1	5.7	53.4	6.9	68,580
45–49	39.5	3.7	54.0	2.8	63,135
Education level*					
No Education	45.9	5.1	43.0	6.0	1,64,986
Primary	41.7	5.5	42.9	9.9	71,376
Secondary	46.9	6.2	32.9	14.0	2,12,569
Higher	52.9	6.6	19.2	21.3	50,695
Place of residence*					
Urban	42.8	5.9	36.0	15.3	166,944
Rural	48.3	5.7	36.4	9.6	332,683
**Wealth index**					
Poor	52.8	5.9	32.6	8.7	189,316
Middle	44.2	5.6	40.5	9.8	102,278
Rich	41.8	5.7	37.5	14.9	208,033
Caste*					
SC	45.1	5.7	38.8	10.4	101,028
ST	50.6	4.3	37.1	8.0	45,537
OBC	48.5	5.0	37.5	9.0	217,677
Others	42.8	7.5	32.1	17.6	134,685
Religion*					
Hindu	45.6	5.6	38.5	10.3	406,897
Muslim	54.7	7.3	20.9	17.0	65,761
Christian	48.8	3.3	40.5	7.5	11,110
Others	33.6	6.0	38.9	21.5	15,859
Region*					
North	38.1	6.6	35.8	19.4	67,049
South	43.3	0.7	53.6	2.4	115,463
East	51.8	7.6	26.3	14.4	115,438
West	41.5	2.7	45.1	10.7	72,022
Central	51.7	9.3	27.4	11.6	112,719
Northeast	51.0	14.1	9.9	25.1	16,936

Note: Traditional methods = Rhythm, Abstinence, Withdrawal; Permanent Methods = Male/ Female sterilization; Reversible modern method = IUCD, injectables, pills, condoms, SDM, LAM.

* Significant association between independent variables and radio listening (Chi-square test, p<0.001)

### Exposure to mass media, messages on FP, and FP use

A higher percentage of current contraceptive use was found among those currently married women of 15–49 years who were either exposed to only television (60%) or exposed to television in combination with other mass media channels (54%) (Table 3). Whereas, the percentage of current non-users was 58% when the women were not exposed to any mass media. Of the women who were exposed only to TV, 11% were using reversible modern methods while 44% were using permanent methods. Of the women who were exposed to TV along with other mass media, 15% used reversible modern methods. It is noteworthy, that for women who were exposed to media other than TV, the percentage of current non-users was high (61%).

**Table 3:** Current use of family planning methods by exposure to mass media and exposure to family planning messages among married women aged 15–49 years in India, NFHS 2015–16.

	Family planning use				
	Non-users	Traditional methods	Permanent methods	Reversible modern methods	Total (N)
	%	%	%	%	
Mass media exposure*					
Not exposed	57.8	6.0	29.2	7.0	104,422
Only TV	39.7	5.8	43.8	10.8	191,082
TV and others	45.8	5.6	33.8	14.9	187,407
Other than TV	61.0	6.0	23.4	9.5	16,716
Exposed to family planning messages*					
No	52.3	5.4	34.3	8.1	146,112
Yes	44.1	5.9	37.1	12.9	353,515

Note: Traditional methods = Rhythm, Abstinence, Withdrawal; Permanent Methods = Male/ Female sterilization; Reversible modern method = IUCD, injectables, pills, condoms, SDM, LAM.

* Significant association between independent variables and radio listening (Chi-square test, p<0.001)

### Spatial variations of dependent and independent variables

**Fig 2:**
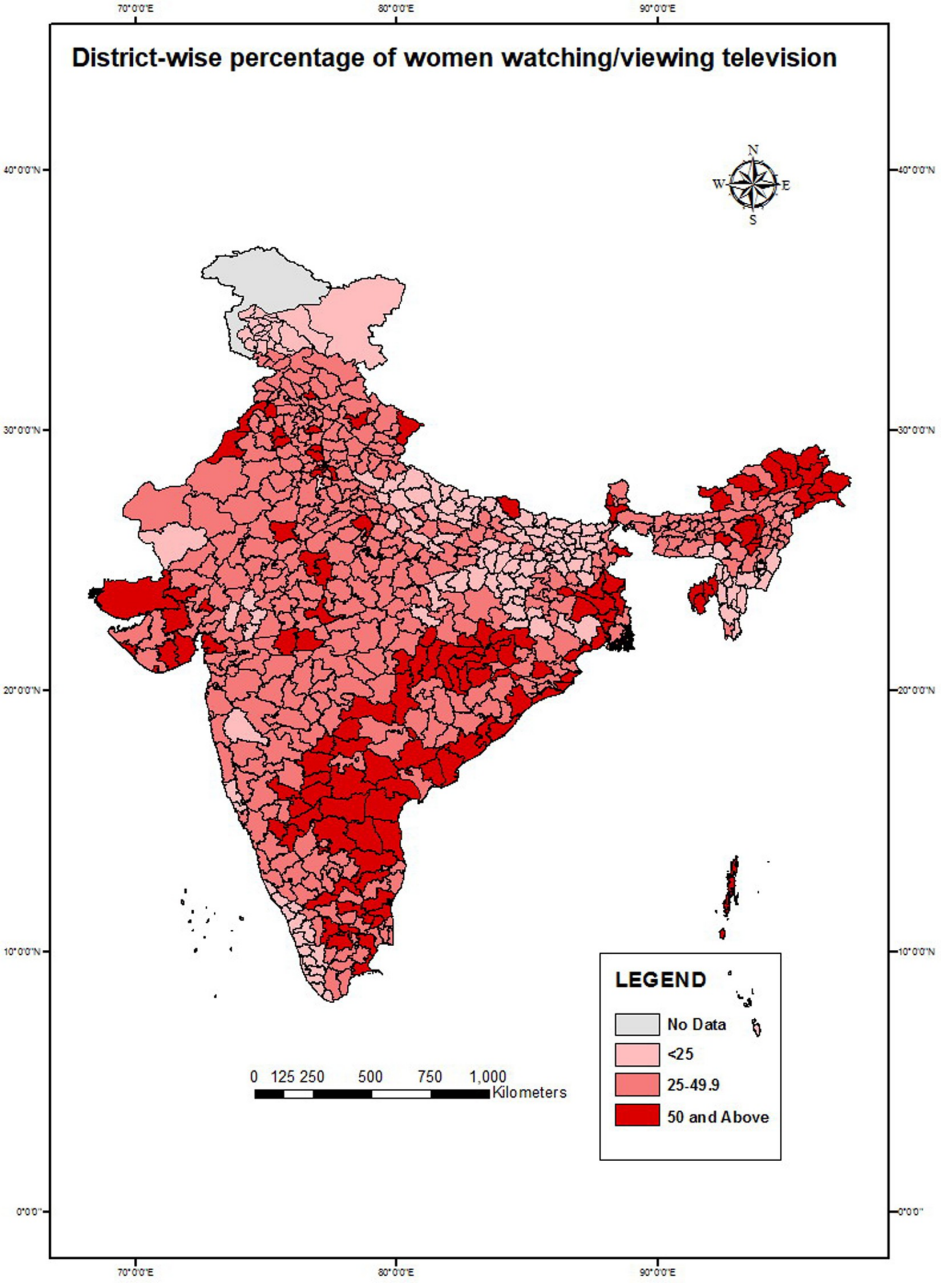
Percentage of married women aged 15–49 years watching/viewing television, India, NFHS 2015–16.

**Fig 3:**
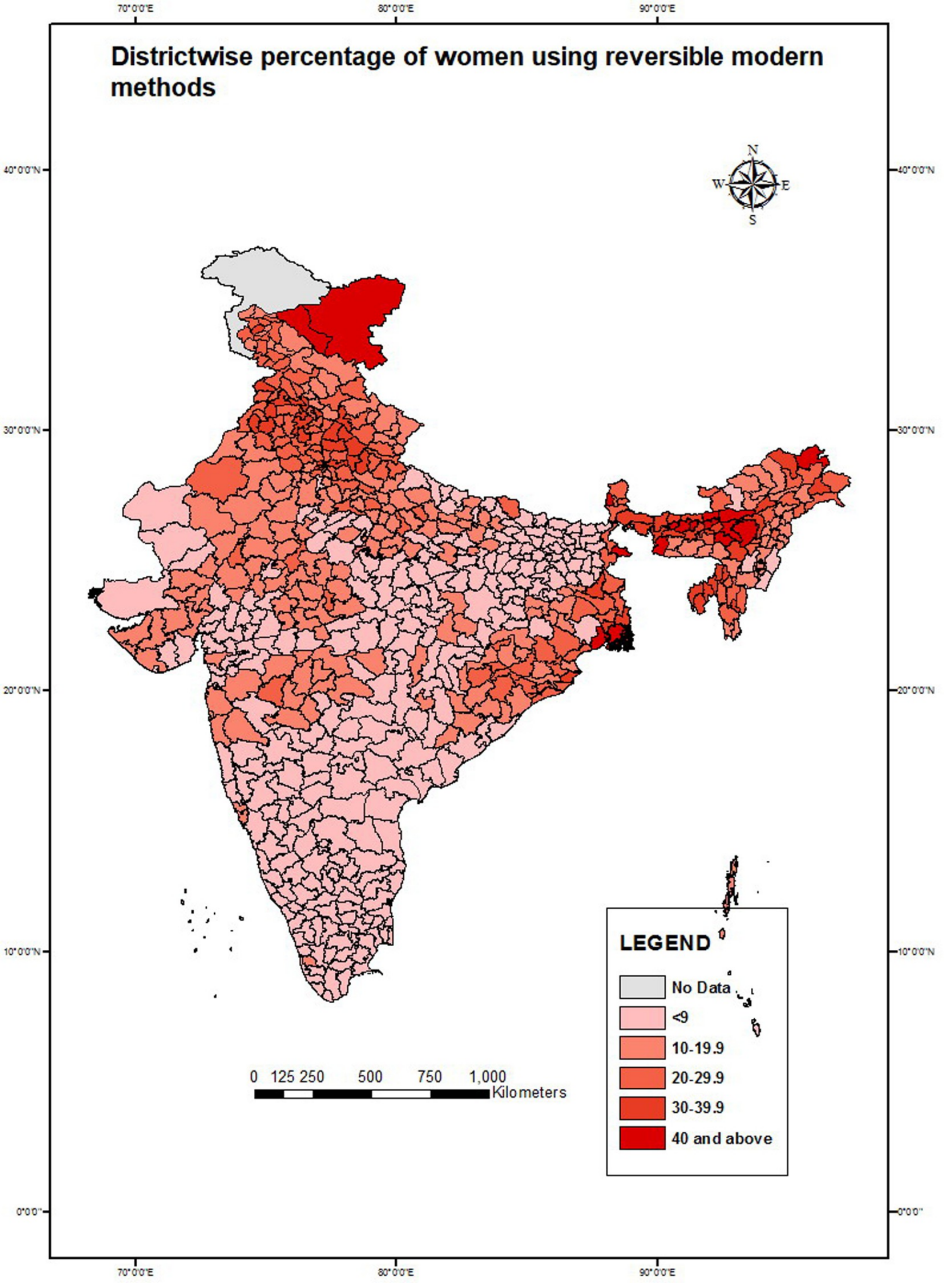
Percentage of married women aged 15–49 years* using reversible modern methods in India, NFHS 2015–16. *Women who underwent sterilization, had a hysterectomy, or declared menopause were excluded.

**Fig 4:**
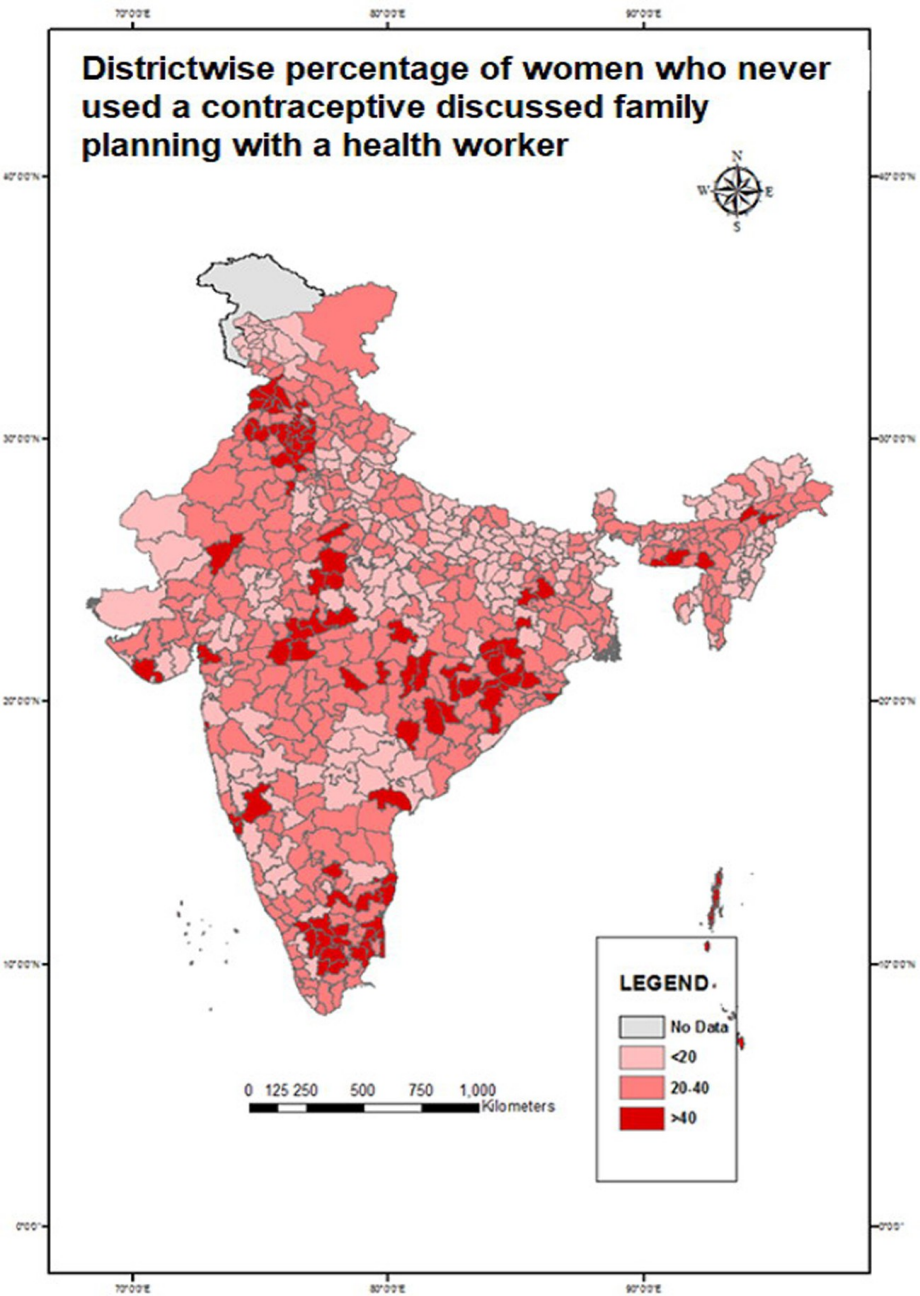
Percentage of married women aged 15–49 years who never used a contraceptive, discussed family planning with a health worker in India, NFHS 2015–16.

**Fig 5:**
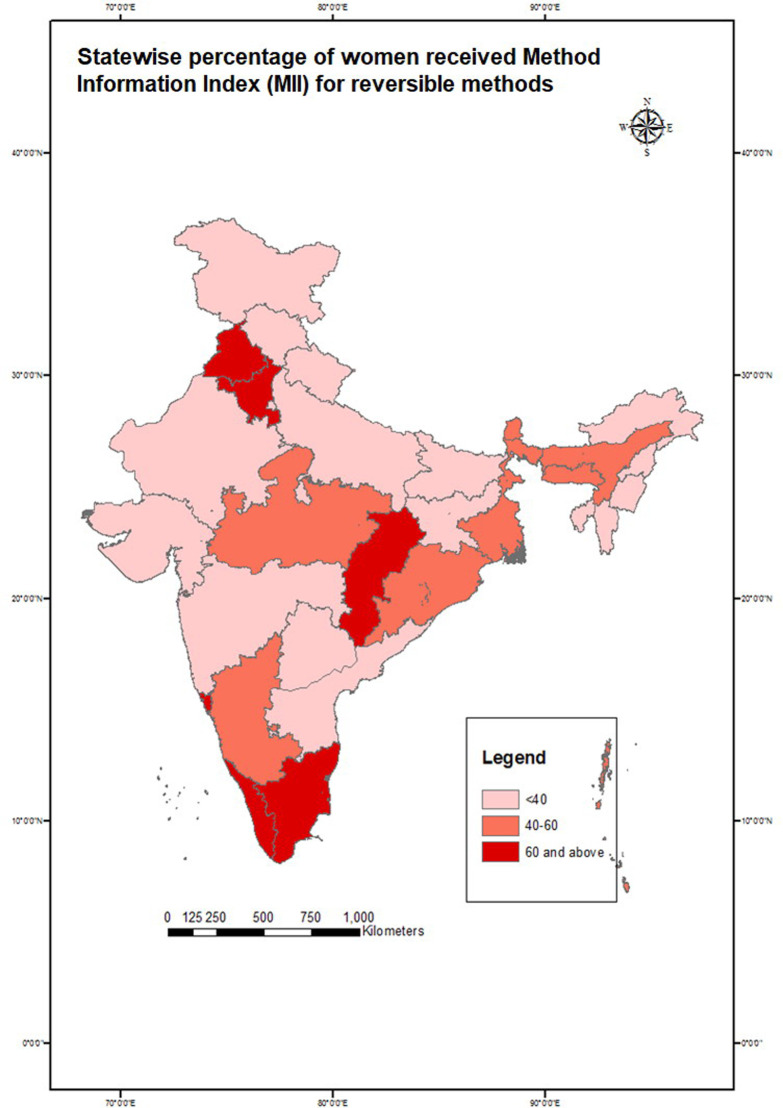
Method information index (in percentage) among married women aged 15–49 years who are currently using reversible modern contraceptives in India, NFHS 2015–16.

Variations of dependent and selected independent variables across different geographies of India were shown with the help of choropleth maps; the district-wise percentage of married women exposed to television (Fig 2), current use of reversible modern methods (Fig 3), discussion on FP by health workers with never-users (Fig 4), and state-wise variation of MII among reversible modern method users (Fig 5). In the maps, the lighter shades indicate a range with a lower percentage while the darker shades indicate a range with a higher percentage.

The choropleth map in Fig 2 shows that most of the districts in the states of Bihar, Uttar Pradesh, Jammu and Kashmir, Rajasthan, and some districts in Northeastern India had low exposure to television among married women of reproductive age (less than 25%). In the southern part of the country, all the districts of Kerala also had low exposure (<25%) to television. Districts of Andhra Pradesh, Arunachal Pradesh, and Gujarat had high exposure to television (more than 50%). Katihar district in Bihar had the lowest exposure (7%), while Tirap district in Arunachal Pradesh had the highest exposure (74%). Most of the districts had a 25%–49% exposure to television.

Fig 3 shows the spatial variation of the current use of reversible modern methods among married women who were fecund and not sterilized. The district-wise choropleth map shows that in most states, especially in the southern and some parts of northern and western parts of India, the current use of reversible modern methods was low (less than 9%), indicating a lower preference for such methods in those areas. The current use of reversible modern methods was high in the eastern parts of the country, mainly in West Bengal. The current use of reversible modern methods was also high (more than 30%) in the northeastern states and some states in Northern India. Kurnool district of Andhra Pradesh and Yadgir district of Karnataka had the lowest percentage of current users of the reversible modern methods while the district of Leh in Jammu and Kashmir and South Twenty-Four Parganas in West Bengal had the highest percentage of women (49%) currently using reversible modern methods.

District-wise percentage of health workers’ coverage on FP is shown in Fig 4. The coverage indicator was calculated by the percentage of women who never used a contraceptive but discussed FP methods with a health worker. Most of the districts in Northern states such as Uttar Pradesh, Madhya Pradesh, Jharkhand, and Bihar had a low coverage of 20%. There were other states with low coverage, which included parts of Gujarat, Rajasthan, Telangana, Arunachal Pradesh, Nagaland, and Manipur. Pulwama district in Jammu and Kashmir had the lowest coverage of 3%, followed by Mon district in Nagaland which had a coverage of 4%. Most of the districts had medium coverage within 20%–40%. Muktsar district in Punjab had the highest coverage of 80%.

The state-wise percentage of MII for reversible methods is shown in Fig 5. Most of the states—especially high focused states under the National Health Mission—had MII of less than 40% which means that about 4 out of ten users of reversible modern contraceptives were not informed fully on the types of methods that could be used, about the side effects of the selected method, or what can be done if side effects were experienced. MII ranged between 40%–60% in Karnataka, Madhya Pradesh, Orissa, West Bengal, Assam, and Meghalaya. MIIs were 60% or higher among users in Kerala, Tamil Nadu, Chhattisgarh, Haryana, Punjab, Goa, and Delhi. Thus, users of reversible modern method in these states were able to make better informed choices of FP methods.

### Decomposition analysis

Table 4 shows the results of Fairlie decomposition analysis, examining the contribution of mass media exposure to the current use of reversible modern methods among married Indian women. A 13 percentage points higher current use of reversible modern methods was found among women exposed to mass media even after controlling for background characteristics of the individuals. Among other independent variables, wealth index and education had significant contributions to the increase in the current use of reversible modern methods. The decomposition analysis also showed that the model can explain 39% of the difference in the current use of reversible modern methods between the group of women who were exposed to mass media and those who were not exposed.

**Table 4:** Decomposition of mass media exposure by current use of reversible modern methods among married women aged 15–49 years in India, NFHS 2015–16.

				95% Confidence Interval	
Independent variable	Coefficient	Standard Error	% Contribution	Lower Boundary	Upper Boundary
**Age (>24 years)**	-0.002*	0.000	-4.286	-0.002	-0.002
**Education Qualification (Literate)**	0.017*	0.001	31.995	0.014	0.019
**Place of Residence (Urban)**	0.008*	0.001	15.252	0.007	0.009
**Caste (Others)**	0.006*	0.000	11.058	0.005	0.006
**Religion (non-Hindus)**	-0.001*	0.000	-1.549	-0.001	-0.001
**Wealth Index (Richer & Richest)**	0.033*	0.001	63.887	0.031	0.035
**Number of Sons (1+ son)**	-0.004*	0.000	-8.497	-0.005	-0.004
**Children Ever Born (1+ child)**	-0.004*	0.000	-7.756	-0.004	-0.004
**Number of observations**	2,49,635				
**N of observations G = 0**	193266				
**N of observations G = 1**	56369				
**Pr(Y! = 0G = 0) [a]**	0.278				
**Pr(Y! = 0G = 1) [b]**	0.145				
**Difference [a-b]**	0.133				
**Total explained [c]**	0.052				
% of contribution c/[a-b]*100	39.2				

Note: * p<0.01 Dependent variable- Contraceptive Use (0 = Non- Users and Traditional Method, 1 = Reversible Method); Group Variable- Mass Media Exposure (1 = Unexposed, 0 = Exposed).

### Multilevel analyses

The decomposition analysis, using individual-level variables, did not thoroughly explain the contribution of mass media. Thus, to examine the role of different predictors for the current use of reversible modern methods—operating at multiple levels such as individual, district, state, and geographic regions—multilevel logistic regressions were conducted (Table 5). The model started with the null model considering only the variables identifying the levels but no independent variable. The results of Model-1 found that the odds of using reversible modern methods were significantly high among women who had been exposed to different mass media channels. Among all types of mass media, television played the most significant role in the use of reversible methods. Women who watched only television (AOR 1.40, 95% CI 1.30–1.50) and were exposed to television along with other mass-media (AOR 1.83, 95% CI 1.45–2.32) had higher adjusted-odds of using reversible modern methods than the women who were not exposed to any mass media. Women who were exposed to other forms of mass media had 1.26 adjusted odds (95% CI 1.10–1.44) of using reversible modern methods than women who were not exposed to mass media. Women who were exposed to FP messages also had 1.25 adjusted odds (95% CI 1.15–1.37) of using reversible modern methods than those who were not.

**Table 5:** Multilevel logistic regression analysis showing odds ratio and 95% confidence interval (CI) for family planning method use among those currently using any reversible modern method for all married women aged 15–49 years in India (N = 237505), NFHS 2015–16.

Predictors	Model—0	Model—1#	Model—2##	Model -3###
	Odds ratio (95% CI)	Odds ratio (95% CI)	Odds ratio (95% CI)	Odds ratio (95% CI)
**Individual-level factors**				
***Media related factors***				
*Mass media exposure*				
Not exposed®				
Only TV	-	1.40 (1.30–1.50) **	1.41 (1.30–1.52) **	1.42 (1.30–1.54) **
TV and others	-	1.83 (1.45–2.32) **	1.56 (1.48–1.63) **	1.57 (1.49–1.65) **
Other than TV	-	1.26 (1.10–1.44) **	1.34 (1.15–1.57) **	1.36 (1.16–1.59) **
*Heard family planning messages*				
No®				
Yes	-	1.25 (1.15–1.37) **	1.22 (1.12–1.33) **	1.22 (1.12–1.32) **
***Background characteristics***				
*Age*				
15–24	-	-	0.79 (0.69–0.91) **	0.79 (0.69–0.91) **
25–34	-	-	1.01 (0.87–1.16)	1.01 (0.87–1.16)
35–49®				
*Place of residence*				
Rural®				
Urban	-	-	1.09 (1.05–1.14) **	1.09 (1.05–1.14) **
*Women’s education level*				
No Education®				
Primary	-	-	1.17 (1.06–1.29) **	1.17 (1.06–1.30) **
Secondary	-	-	1.20 (1.10–1.31) **	1.20 (1.10–1.31) **
Higher	-	-	1.47 (1.24–1.76) **	1.49 (1.24–1.78) **
*Husband’s education level*				
No education®				
Primary	-	-	1.14 (1.01–1.29) *	1.15 (1.01–1.30) *
Secondary	-	-	1.20 (1.08–1.34) **	1.21 (1.09–1.34) **
Higher	-	-	1.29 (1.13–1.49) **	1.30 (1.13–1.49) **
*Women’s occupation*				
Unemployed®				
Employed	-	-	1.10 (0.98–1.24)	1.10 (0.98–1.24)
*Husband’s occupation*				
Unemployed®				
Employed	-	-	1.03(0.90–1.18)	1.03(0.90–1.18)
*Husband’s residential status*				
Living with the respondent®				
Staying elsewhere	-	-	0.40 (0.38–0.42) **	0.40 (0.38–0.42) **
*Social group*				
SC®				
ST	-	-	0.94 (0.81–1.08)	0.94 (0.81–1.09)
OBC	-	-	1.01 (0.94–1.09)	1.01 (0.94–1.09)
Others	-	-	1.12 (1.02–1.23) *	1.13 (1.03–1.24) *
*Religion*				
Hindu®				
Muslim	-	-	1.20 (1.06–1.34) **	1.21 (1.08–1.35) **
Christian	-	-	0.96 (0.84–1.09)	0.96 (0.84–1.09)
Others	-	-	1.18 (1.02–1.38) *	1.18(1.01–1.39) *
*Wealth index*				
Poor®				
Middle	-	-	1.17 (1.08–1.27) **	1.18 (1.09–1.28) **
Rich	-	-	1.37 (1.21–1.56) **	1.38 (1.21–1.56) **
***Reproductive history***				
*Children ever born*				
No child®				
One child	-	-	3.73 (3.07–4.54) **	3.79 (3.12–4.59) **
Two children	-	-	5.20 (4.25–6.36) **	5.30 (4.36–6.45) **
Three children	-	-	4.88 (4.12–5.79) **	4.98 (4.22–5.87) **
More than three children	-	-	4.76 (3.83–5.92) **	4.85 (3.94–5.99) **
*Number of sons*				
No son®				
One son	-	-	1.37 (1.21–1.55) **	1.37 (1.21–1.55) **
Two or more sons	-	-	1.50 (1.33–1.71) **	1.51 (1.33–1.72) **
**District-level factor**				
*Percentage of non-users discussed FP with health workers*	-	-	-	1.01 (1.00–1.02) **
**State-level factor**				
*Method information index (in %)*	-	-	-	1.20 (1.06–1.35) **
**Random effects parameters**				
Variance (SE)				
Regional-level	0.34 (0.22)	0.34 (0.24)	0.32 (0.21)	0.37 (0.24)
State-level	0.38(0.15)	0.40 (0.16)	0.37 (0.13)	0.20 (0.05)
District-level	0.22 (0.02)	0.19 (0.02)	0.19 (0.01)	0.19 (0.01)
VPC (PCV in %)				
Regional-level		0.082 (0.00)	0.076 (77.66)	0.091 (73.09)
State-level	-	0.104 (-5.26)	0.095 (74.93)	0.055 (85.53)
District-level	-	0.053 (13.64)	0.054 (75.67)	0.054 (75.54)

Note: ® = Reference category;

** p<0.01

*p<0.05; CI = Confidence Interval; VPC = Variance Partition Coefficient; Proportional Change in Variance

Dependent Variable- Using Any Reversible Method (0 = Non -users and traditional method, 1 = Reversible Modern Method); Sterilization and infecund women were not considered.

Model 0: Null Model; no independent variables were considered for any levels.

#Model 1: Media related variables.

##Model 2 Independent variables include Model 1 + Independent variables include individual level variables i.e., background characteristics + reproductive history + geographic regions.

###Model 3: Independent variables include variables in Model 2 + FP program coverage + Method Information Index

There was no major change in results between Model-1 and Model-2, where the association was further controlled with the background characteristics of the respondents, further showing the significant association between exposure to mass media and current use of reversible modern methods. Model-2 also showed significantly low current use of reversible modern methods among young women (15–24 years) (AOR 0.79), but a significantly higher current use among women living in urban areas (AOR 1.09), with higher education (AOR 1.47), of ‘other’ social-group (AOR 1.12) and religion, and belonging to ‘rich’ wealth group (AOR 1.37). Women whose husbands were living elsewhere had significantly lower adjusted odds of using a reversible modern method (AOR 0.40).

In the context of the reproductive history variable of women who had ‘children ever born’, odds of using reversible modern methods were significantly higher. The adjusted odds of using reversible modern methods were 5.00 among mothers with 2 children, which decreased among women with 3 children (AOR 4.88) or more (AOR 4.76). For women who had 2 or more sons, the adjusted odds for using a reversible modern method were much higher (AOR 1.50) than those who did not have any sons.

In Model-3, the district level factor—health workers who discussed FP with non-users—and the state level factor—the MII—were included along with the variables from the previous models. Significantly higher adjusted odds of current use of reversible modern methods were reported for those women who belonged to districts where a higher percentage of ‘never-users’ discussed FP with health workers. An increase of 1 percentage point in this independent variable resulted in 1.01 times increase in odds for the current use of reversible modern methods. The state-level variable, MII for reversible modern methods, also showed significantly higher odds for the current use of reversible modern methods. A 1 percentage-point increase of MII at the state level could result in 1.2 times increase in odds for the current use of reversible modern methods.

The odds of using reversible modern methods for those who were exposed to mass media remained the same even after adjusting for district-level and state-level variables, and the geographic region. This finding confirmed that mass media exposure had a significant independent effect on the current use of reversible modern methods even after controlling for other determinants of contraceptive use operating at multiple levels.

## Discussion

The study provides important insights into the contribution of mass media exposure to the current use of reversible modern FP methods among married women in India. It is found that accessibility to the information provided by different mass media channels will make women aware of different FP methods. The study further found that among all mass media channels, television was the most effective medium for disseminating FP messages, followed by television in combination with other mass media. The exposure to other forms of mass media was low, which could be because those media channels were less popular among Indian women. Access to mass media and exposure to FP messages were also significantly associated.

By reviewing these findings, it can be deduced that exposure to television plays an important role in FP use among Indian women. It was not possible to compare the results of this study with other similar studies due to the lack of literature on this issue in India. However, some earlier studies in other countries like Kenya, Nigeria, Senegal, Malawi, Burkina Faso, Nepal, and Bangladesh showed that exposure to television had a strong effect on the likelihood of contraceptive use [7, 11, 15, 19–21]. Some studies in Sub-Saharan Africa also found an association of mass media exposure with contraceptive use [4, 7]. Though, these studies did not focus on multilevel analyses based on which could provide a clearer understanding about the factors working at different levels and how mass media exposure plays a significant role in increasing the use of modern FP methods.

The association of other background characteristics with reversible method use showed higher method use among younger women than older women. This could be because young women have not achieved their desired goal yet in terms of the number of children they wish to have, motivating them to use reversible methods instead of permanent methods. Higher current use of reversible modern methods has been reported among women with higher education, of ‘other’ social groups, and belong to ‘rich’ households. The higher use of reversible modern methods among women with sons highlights the presence of son preference in India; a woman or her family desires at least one son, after which her use of contraceptive methods increases. A similar study on religion, contraceptive method mix, and son preference among the Bengali-speaking community in the Indian subcontinent found greater similarity in contraceptive behaviors for son preference among Hindus of Eastern India and Bangladesh, as well as the Bangladeshi Muslims. While Muslims of Eastern India were more inclined toward keeping the family size large and were less concerned with the sex composition of the family [21].

The study also highlighted spatial variations across states and districts with regards to the association between being exposed to mass media and reversible modern method use. Several geographical pockets were identified as having less exposure to television, such as the high focus states in the northern part of India, north-eastern states, and some parts of Southern India. On the contrary, women from most of the districts in the northern belt of Uttar Pradesh and Bihar, Western India, and most of Southern India had a low prevalence of use of reversible modern methods. These findings highlight the prevalent regional disparities in the current use of reversible modern methods and mass media exposure which need to be addressed programmatically, especially in the northern and western parts of the country.

The findings of the decomposition analysis demonstrated that mass media exposure significantly contributed to the variation in the current use of reversible modern methods, which was around 49% after controlling for other predictors. The odds ratios from the multilevel analyses also suggested that the current use of reversible modern methods increased with an increase in the frequency of mass media exposure and hearing FP messages through mass media as well as other demographic, socio-economic, and spatial predictors.

The study found that a higher coverage by the health workers showed a strong association with reversible modern contraceptive use. An earlier study by Gupta et al. also reported a corroborative finding that health workers and mass media played very important roles in disseminating information about the promotion of contraceptive practices and can help overcome the knowledge-practice gap [17]. This study also demonstrated the association of better-informed choice, measured in terms of MII, with higher use of reversible modern contraceptives. The finding is critical when the national FP program is emphasizing on ensuring access to reversible methods for voluntary use of contraceptives.

This study has some limitations. The NFHS provides information on the frequency of mass media exposure, which does not give information about the types of programs watched or heard. The analyses would have been more comprehensive if these limitations had been addressed during survey design and data collection.

### Programmatic recommendations

Mass media exposure for FP should be increased as it is a cost-effective medium to raise awareness and share information among women. The evidence generated through this study suggests that the exposure to FP messages in mass media has a strong determining factor for the use of reversible modern methods. This is critically important because the government has shifted its focus from permanent methods to reversible methods, especially among zero and low-parity women [22]. Therefore, media coverage, as well as the accessibility of mass media, should be improved in the districts with less exposure to mass media. Such development will increase exposure to FP-related messages in those areas as well. Alternative arrangements should be made in the ‘media dark areas’ of Northern India, Northeastern India, and the discordant pockets by involving more health workers, NGOs, and women self-help groups. Media exposure to FP programs in national as well as private channels is necessary because television viewing showed a significant association with the FP method used.

## Conclusions

This study highlights that mass media exposure has a strong effect on FP use, even after controlling for other individual-level and contextual variables, and therefore, the findings can be used as evidence of the utility of this medium to inform the masses on FP in a cost-effective manner. At the same time, the findings from the multilevel analyses suggest that media exposure coupled with programmatic efforts like coverage of the target population by health care workers, and the receiving of informed choices can synergistically increase the use of reversible methods. The findings of this study also demonstrate a considerable spatial variation in mass media exposure and its association with FP use. This suggests the importance of geography-specific programs with a targeted approach to reach different population segments for better implementation of the FP program in India and make the FP services more accessible to the women who are in most need.

## Supporting information

S1 Fig Step by step exclusion of the sample to derive the final sample for analysis. (TIF)

S1 Table List of variables in the analysis and the recoded categories. (DOCX)

S2 Table Ownership of mass media and exposure to family planning messages on mass media (in percentage) among married women aged 15–49 years in India, NFHS 2015–16 (N = 481,512). (DOCX)
