# Supplementary figures and images for: Mass media exposure and use of reversible modern contraceptives among married women in India: An analysis of the NFHS 2015–16 data
Source: PLoS One. 2021 Jul 13;16(7):e0254400. doi: 10.1371/journal.pone.0254400 (PMC8277022; doi:10.1371/journal.pone.0254400)

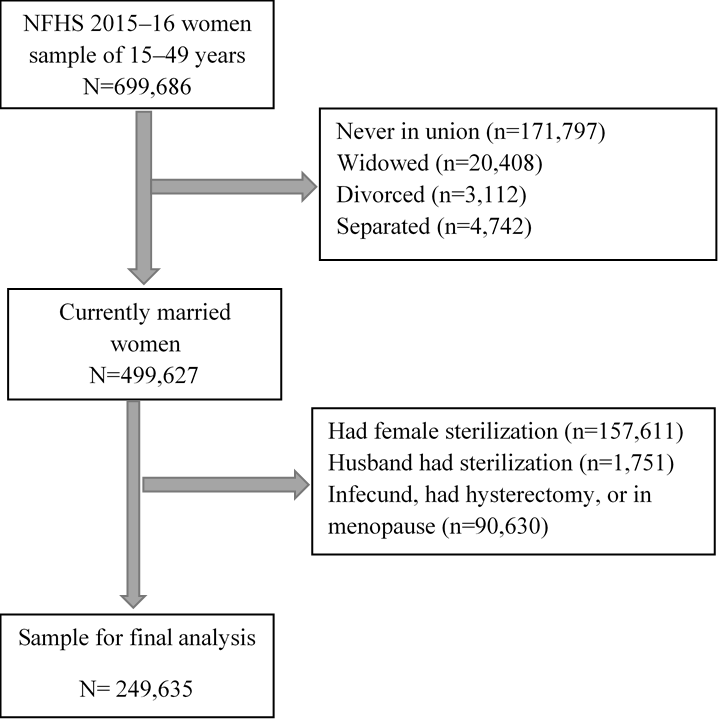

Supplement: S1 Fig — (TIF) [file pone.0254400.s001.tif]
