# Supplementary material for: Mass media exposure and use of reversible modern contraceptives among married women in India: An analysis of the NFHS 2015–16 data
Source: PLoS One. 2021 Jul 13;16(7):e0254400. doi: 10.1371/journal.pone.0254400 (PMC8277022; doi:10.1371/journal.pone.0254400)
Supplement: S1 Table — (DOCX) [file pone.0254400.s002.docx]

S1 Table. List of variables in the analysis and the recoded categories.

| **List of variables** | **Recoded categories** |
| --- | --- |
| Mass media exposure | 0 ‘Not exposed’ 1 ‘Only TV’ 2 ‘TV and others’ 3 ‘Other than TV’ |
| Heard family planning messages | 0 ‘No’ 1 ‘Yes’ |
| Age group | 1 ‘15-24 years’ 2 ‘25-34 years’ 3 ’35-49 years’ |
| Place of residence | 1 ‘Rural’ 2 ‘Urban’ |
| Women’s education level | 0 ‘No Education’ 1 ‘Primary’ 2 ‘Secondary’ 3 ‘Higher’ |
| Husband’s education level | 0 ‘No Education’ 1 ‘Primary’ 2 ‘Secondary’ 3 ‘Higher’ |
| Women’s Occupation | 0 ‘Unemployed’ 1 ‘Employed’ |
| Husband’s occupation | 0 ‘Unemployed’ 1 ‘Employed’ |
| Husband’s residential status | 1 ‘Living with the respondent’ 2 ‘Staying elsewhere’ |
| Social groups ^1^ | 1 ‘Scheduled caste (SC)’ 2 ‘Scheduled tribe (ST)’ 3 ‘Other backward classes (OBC)’ 4 ‘Others’ |
| Religion | 1 ‘Hindu’ 2 ‘Muslim’ 3 ‘Christian’ 4 ‘Others’ |
| Wealth index | 1 ‘Poor’ 2 ‘Middle’ 3 ‘Rich’ |
| Children ever born | 0 ‘No Child’ 1 ‘One child’ 2 ‘Two children’ 3 ‘Three children’ 4 ‘More than 3 children’ |
| Number of sons | 0 ‘No son’ 1 ‘One son’ 2 ‘Two or more sons’ |
| Region | 1 ‘North’ (Chandigarh, Haryana, Himachal Pradesh, Jammu and Kashmir, Delhi, Punjab, Rajasthan, Uttarakhand)  2 ‘South’ (Andaman and Nicobar Islands, Andhra Pradesh, Karnataka, Kerala, Lakshadweep, Puducherry, Tamil Nadu, Telangana)  3 ‘East’ (Bihar, Jharkhand, Odisha, West Bengal)  4 ‘West’ (Dadra and Nagar Haveli, Daman and Diu, Goa, Gujarat, Maharashtra)  5 ‘Central’ (Chhattisgarh, Madhya Pradesh, Uttar Pradesh)  6 ‘Northeast’ (Arunachal Pradesh, Assam, Manipur, Meghalaya, Mizoram, Nagaland, Sikkim, Tripura) |

^1 Scheduled caste (SC), scheduled tribes (ST), and other backward classes (OBC) are marginalized groups in India designated by the government and recognized by the Constitution of India^
