# Supplementary material for: Mass media exposure and use of reversible modern contraceptives among married women in India: An analysis of the NFHS 2015–16 data
Source: PLoS One. 2021 Jul 13;16(7):e0254400. doi: 10.1371/journal.pone.0254400 (PMC8277022; doi:10.1371/journal.pone.0254400)
Supplement: S2 Table — (DOCX) [file pone.0254400.s003.docx]

S2 Table. Ownership of mass media and exposure to family planning messages on mass media (in percentage) among married women aged 15–49 years in India, NFHS 2015–16 (N=481,512).

| **Mass media exposure** | **Exposure to family planning messages on mass media** |
| --- | --- |
| Households exposed to no mass media and family planning messages | 21.2 |
| Households with radio | 2.4 |
| Households with television | 57.1 |
| Households with both radio and television | 19.4 |

Note: Non-de jure residents were excluded
